# The Hertfordshire Cohort Study: an overview

**DOI:** 10.12688/f1000research.17457.1

**Published:** 2019-01-21

**Authors:** Holly E. Syddall, Shirley J. Simmonds, Sarah A. Carter, Sian M. Robinson, Elaine M. Dennison, Cyrus Cooper, The Hertfordshire Cohort Study Research Group

**Affiliations:** 1 MRC Lifecourse Epidemiology Unit, Southampton, Hampshire, SO16 6YD, UK; 2 NIHR Southampton Biomedical Research Centre, University of Southampton, Southampton, Hampshire, SO16 6YD, UK; 3 NIHR Musculoskeletal Biomedical Research Unit, University of Oxford, Oxford, Oxfordshire, OX3 7LD, UK

**Keywords:** Hertfordshire, Cohort Study, Epidemiology, Ageing, Musculoskeletal, Lifecourse

## Abstract

The Hertfordshire Cohort Study is a nationally unique study of men and women born in the English county of Hertfordshire in the early part of the 20 ^th^ century. Records that detail their health in infancy and childhood have been preserved, their sociodemographic, lifestyle, medical and biological attributes have been characterised in later life, and routinely collected data on their hospital use and mortality have been acquired. This paper provides an overview of the study since its inception in the 1980s, including its methods, findings, and plans for its future.

## Background to the Hertfordshire Cohort Study

In the 1980s, ecological studies showed that death rates from heart disease in the 212 local authority areas of England and Wales during 1968–78 were correlated with infant mortality rates in those areas in the early part of the 20 ^th^ century ^1^. This raised the possibility that environmental influences acting during the fetal and infant stages of development might increase risk of cardiovascular disease in later life.

However, stronger evidence from a more robust epidemiological study design was required. The discovery of a large set of birth records for people born in Hertfordshire during the first half of the twentieth century provided an opportunity for the MRC Environmental Epidemiology Unit (MRC EEU) to establish a cohort study in which the early origins of disease in later life could be explored.

### The Hertfordshire birth records

**Figure 1.**
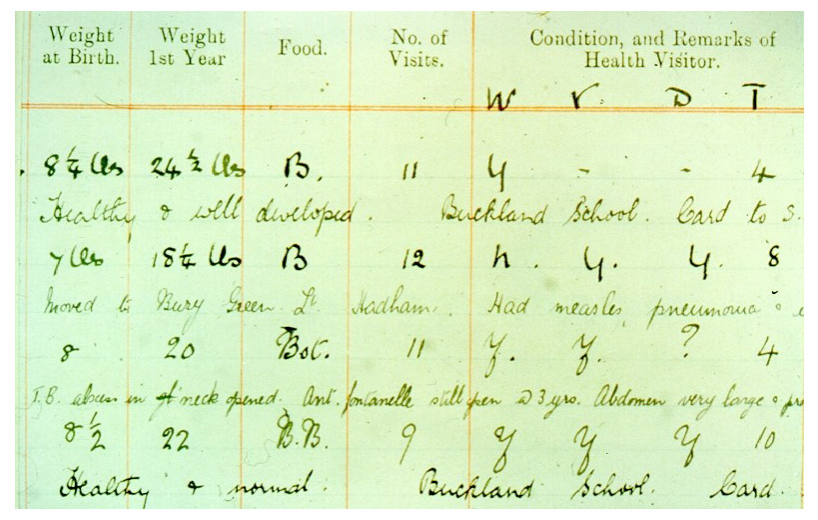
An extract from the Hertfordshire ledgers. Source: original Hertfordshire ledger, on loan from the Hertfordshire Local Studies Archive; currently stored securely at the MRC LEU.

In 1911, a team of midwives and nurses was assembled in Hertfordshire with the aim of improving the health of children in the county. Midwives attended women during childbirth and health visitors routinely visited the child throughout infancy and childhood; weight at birth and one year of age, method of infant feeding, illnesses, and developmental milestones were all recorded. The information ( Table 1) was transcribed into ledgers ( Figure 1) at the County Health Department in Hertford. The ledgers cover almost all births in Hertfordshire from 1911 until the NHS was formed in 1948.

**Table 1.** Hertfordshire Health Visitors’ ledgers.

**Key data availability:**
*At birth* • Name and address • Date of birth • Gestation • Sex • Weight
*During infancy (1 ^st^ year of life)* • Method of feeding • Whether given a dummy • General comments on health
*At first birthday* • Weight • Whether weaned • Whether vaccinated (availability of disease-specific vaccines varied by birth year) • Number of teeth
*From age 1–5 years* • General comments on health

### Studies based on men and women born in Hertfordshire 1911–30

The MRC EEU computerised the Hertfordshire ledgers and traced the cohort by linkage with the National Health Service Central Register (NHSCR). A mortality study of 15,000 men and women born between 1911–30 showed that lower weight at birth and one year of age was associated with increased risk of death from cardiovascular disease by 75 years of age ^2^. Subsequently, numerous detailed physiological studies were conducted among community dwelling men and women born in Hertfordshire between 1920–30 and still living there in the early 1990s. Small size in early life was associated with greater risk of cardiometabolic disease ^3– 5^, osteoporosis ^6^ and sarcopenia (the loss of muscle mass, strength and function with age) ^7^ in later life. Collectively these studies contributed to the “developmental origins” hypothesis which suggests that the nourishment a baby receives in utero, and its development in infancy and early childhood, determine its risk of disease in later life ^8^.

These early studies were important for establishing relationships between the early environment and physiological markers of disease, but they lacked detailed information on adult anthropometry and diet, and the men and women born 1920–30 were becoming frail and unable to take part in further studies. A second (younger) cohort, in whom the work could be continued, was therefore recruited between 1998–2004. During this time Professor Cyrus Cooper became Hertfordshire Cohort Study (HCS) principal investigator and the MRC EEU was reconfigured as the MRC Lifecourse Epidemiology Unit (LEU), a University Unit Partnership between the MRC and the University of Southampton.

## Key methods

The younger cohort comprises 3000 men and women born between 1931 and 1939 who were traced following the model of the early studies. These are the people to whom the term ‘The Hertfordshire Cohort’ specifically applies. The remainder of this paper applies only to them, unless otherwise stated.

### Objective of the Hertfordshire Cohort Study

The principal objective of the HCS is to evaluate interactions between the intra-uterine and early postnatal environment, adult diet and lifestyle, and genetics, in the aetiology of chronic disorders of ageing (osteoporosis, osteoarthritis, sarcopenia, obesity, cardiometabolic disease and type II diabetes mellitus). The study aims to place these interactions within a life-course model for disease pathogenesis, and to characterise the physiological mechanisms underlying the pathways to these chronic disorders.

### Baseline recruitment 1998–2004

**Figure 2.**
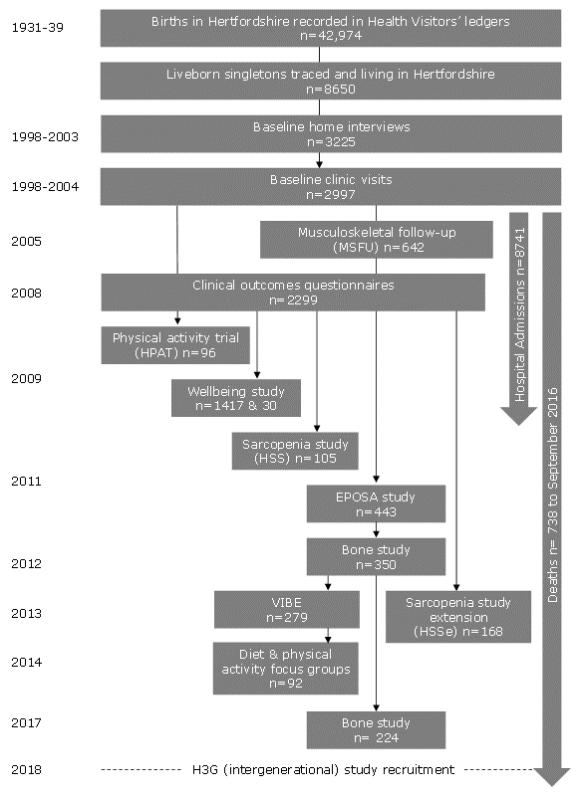
Participant recruitment and summary of studies carried out from 1998–2018.

The baseline recruitment of HCS participants has been described in detail previously ^9^; key stages in the process are shown in Figure 2. In brief, the ledgers contained records for 42974 births in Hertfordshire between 1931 and 1939; the National Health Service Central Register (NHSCR) in Southport (now part of NHS Digital) traced 8650 men and women who were still alive in Hertfordshire in 1998. Permission to contact 6099 men and women by letter was obtained from their GPs and 3225 (53%) agreed to a home interview with a trained research nurse. 2997 (93%) men and women subsequently attended a clinic for detailed physiological investigations. The baseline HCS fieldwork was county-wide but conducted in phases by geographical area and gender. 966 (68%) of the 1412 men and women who attended clinic in East Hertfordshire also underwent a Dual-energy X-ray Absorptiometry (DXA) bone scan and knee radiography. An overview of the data collected at the HCS baseline home interviews and clinics is provided in Table 2.

**Table 2.** Hertfordshire Cohort Study Baseline.

Key data availability	
Home interview	Clinic Visit
• Marital status • Age left full-time education • Housing tenure • Car availability • Family history including father’s social class • Dallosso physical activity questionnaire • Cigarette smoking • Alcohol consumption • Obstetric history • Occupational history • Current Social class • Rose/WHO chest pain and leg pain questionnaires • Severe chest pain and previous coronary surgery • Respiratory symptoms (MRC questionnaire) • Fracture history (own and of parents and siblings) • Lower back pain • Medical history (including stroke and diabetes) • Current medications • Falls • Self-rated general health • SF-36 health related quality of life • Anxiety and depression (HAD) scores • Current diet (administered food frequency questionnaire and 24-hour food diary) • Nutrient intake from dietary supplements • Social support and networks • Job effort-reward and demand-control	• Height; weight; waist, hip, mid-upper arm and thigh circumferences • Triceps, biceps, subscapular and suprailiac skinfold thicknesses (Harpenden callipers) • Blood pressure and pulse rate (Dinamap recorder) • Lung function FEV1 and FVC (Micro Spirometer, Micro Medical) • Standard 12-lead electrocardiography • Venous blood samples after overnight fast: Glucose Insulin and proinsulin precursors Total, HDL and LDL cholesterol Triglycerides Apolipoprotein A1 and B Vitamin C • Frozen plasma and sera stored for future measurements • Two-hour timed 75g oral glucose tolerance test: Glucose and insulin 30’ and 120’ post load • DNA extracted from whole blood samples • Timed overnight urine collection • Grip strength (Jamar hand-grip dynamometer) • Quadriceps strength (West Hertfordshire only, Lafayette MMT strength system) • Timed 6m up-and-go test and 3m walk • Chair rises • Timed one-legged stand • Clinical hand examination for pain, swelling and tenderness • DXA scan of lumbar spine and femoral neck (Hologic QDR 4500) • Antero-posterior and lateral x-rays of both knees (East Hertfordshire only)

### Ethical permission and informed consent

The HCS baseline investigations had ethical approval from the Hertfordshire and Bedfordshire Local Research Ethics Committee and all subjects gave written informed consent. All interviews and physiological investigations were carried out according to strict protocols and studies of within- and between-observer variation were conducted at regular intervals during fieldwork to ensure comparability of measurements obtained over time. Data were collected, anonymised, processed and stored in accordance with contemporary data protection regulations. Participants are free to withdraw from the study at any time.

### Follow-up studies 2004 to 2018

Since their first contact in 1998–2004, HCS cohort members have generously agreed to take part in various follow-up postal questionnaires, face-to-face interviews, clinics, intervention studies, and focus groups (see Figure 2). With their consent, data on hospital admissions experienced by the cohort members across a decade have been obtained from NHS Digital ^10^, and cohort members remain flagged on the National Health Service Central Register for ongoing notification of mortality. Ethical approval was obtained for all elements of the study at the time they were conducted.

Table 3 to Table 11 describe the scientific objective and methodology of each HCS follow-up study and detail key data availability. Taken together, the HCS datasets provide a rich, detailed and longitudinal characterisation of the sociodemographic, lifestyle, medical and biological attributes of a contemporary group of community-dwelling older men and women in the UK.

**Table 3.** Hertfordshire Cohort Study Musculoskeletal Follow-Up study (MSFU).

**Objective:**	To characterise change in musculoskeletal health over a four year period in later life.
**Methodology:**	Face to face assessments among HCS participants resident in East Hertfordshire who underwent a DXA scan at baseline.
**Key data availability:**	
• Housing tenure • Cigarette smoking • Alcohol consumption • Fracture history (own) • Falls • Strawbridge frailty score • Fried frailty components • Townsend disability scale • General bodily pain questionnaire • Medical conditions diagnosed by doctor since HCS baseline • Hospital admissions since HCS baseline • Current medications • SF-36 health related quality of life • Anxiety and depression (HAD) scores • Height; weight; waist, hip, mid-upper arm and thigh circumferences • Triceps, biceps, subscapular & suprailiac skinfold thicknesses Harpenden callipers) • Grip strength (Jamar hand-grip dynamometer) • Timed 6m up-and-go test and 3m walk • Chair rises • Timed one-legged stand • Quadriceps strength (Lafayette MMT strength system) • Clinical hand examination for pain, swelling and tenderness • Radial and tibial pQCT scans (Stratec XCT 2000XL instrument) • DXA scan of lumbar spine and femoral neck (Hologic QDR 4500)	

## Key findings

HCS is a particularly valuable resource for research that aims to identify lifecourse influences on ageing. Longitudinal measurements are available for a range of markers of disease and for potential predictor and confounding variables. The study has been pioneering in its uptake of techniques not previously used in epidemiology to characterise health (for example High Resolution peripheral Quantitative Computed Tomography (HR pQCT) scanning and muscle biopsy) and has sought to maximise the value of routinely collected data through record linkage.

The following sections outline key publications in the areas of osteoporosis, osteoarthritis, sarcopenia and diet.

### Osteoporosis

Osteoporosis is the most common metabolic bone disease affecting older people; almost one in two women and one in five men aged 50 years will have an osteoporotic fracture in their remaining lifetime ^11^. The LEU is a renowned contributor to research on osteoporosis, in part because of the wealth of data collected in HCS ( Table 2– Table 12). Estimates of bone mineral density (BMD) and bone mineral content (BMC) have been obtained by DXA; estimates of bone strength (including strength strain index (SSI)), cortical bone and trabecular bone by peripheral Quantitative Computed Tomography (pQCT); and ‘virtual bone biopsies’ by HR pQCT. Few other cohorts have characterised bone in such detail.

**Table 4.** Hertfordshire Cohort Study clinical outcomes questionnaire.

**Objective:**	To ascertain information on medical diagnoses and clinical events experienced by cohort members since baseline.
**Methodology:**	Cohort wide postal questionnaire.
**Key data availability:**	
• Falls • Fractures • Strawbridge frailty score • Fried frailty self-reported components • Townsend disability scale • General bodily pain questionnaire • WOMAC knee pain questionnaire • Clinical conditions diagnosed by a doctor (including: hypertension; diabetes; respiratory illness; rheumatoid arthritis; multiple sclerosis; thyroid disease; vitiligo; depression; Parkinsons disease; cancer; other serious illnesses) • Hospital admissions (date and cause) • History of coronary tests and procedures (including treadmill exercise tests, angiogram/x-ray, angioplasty of coronary arteries, and coronary artery bypass graft) • Symptoms of stroke/transient ischaemic attack • Current medications	

**Table 5.** Hertfordshire Physical Activity Trial (HPAT).

**Objective:**	To examine whether the impact of aerobic exercise training on metabolic risk, physical fitness and function, and body composition differs with respect to growth in early life.
**Methodology:**	Randomised controlled trial conducted in collaboration with University of Cambridge. 100 HCS participants randomised to exercise-intervention (12 week gym training programme) or placebo (habitual physical activity) arm of the trial. Assessments conducted at HPAT baseline and 12 week follow-up ^20^.
**Key data availability:**	
• Blood pressure (Omron recorder) • Standard 12-lead electrocardiography • Two-hour timed 75g oral glucose tolerance test: Glucose and insulin 30’ and 120’ post load • Venous blood samples after overnight fast: Glucose Insulin Liver and lipid profiles C-peptide • Height; weight; waist and hip circumferences • Whole body DXA scan (Lunar Prodigy Advanced scanner) • Ultrasound measurement of body composition (LOGIQ Book XP device) • MRI scans of liver and muscle composition (Siemens 3T Tim Trio scanner) • Sub-maximal cycle ergometry exercise test (heart rate, energy expenditure and oxygen consumption) • 7-day Actiheart physical activity monitoring • Physical activity questionnaire (modified EPAQ2) • Grip strength (Jamar hand-grip dynamometer) • Timed 6m up-and-go test and 3m walk • Chair rises • Timed one-legged stand	

**Table 6.** Hertfordshire Cohort Study wellbeing study.

**Objective:**	To explore lifecourse influences on wellbeing in older people.
**Methodology:**	Principal questionnaire sent by post; a subset of participants completed a qualitative interview by telephone. This study was a component of the HALCyon (Healthy Ageing across the Lifecourse) collaborative research project, directed by the MRC Unit for Lifelong Health and Ageing at UCL and originally funded by the New Dynamics of Ageing (NDA) programme.
**Key data availability:**	
• Neighbourhood cohesion score • Warwick Edinburgh mental wellbeing scale • IPIP (International Personality Item Pool) 5-factor 58-item personality scale (extraversion, emotional stability, conscientiousness, agreeableness and openness to experience) • Diener 5-item life satisfaction scale • Rand 18-item social support scale (scores coded overall and for emotional support, affectionate support, tangible support and positive interaction) • Nottingham Activity and Ageing Study 9-item social engagement scale • Income (type and amount) • Perceptions of problems with housing • Perceptions of neighbourhood problems • Falls • Townsend disability scale • Hospital admissions since age 65 • Services used	

**Table 7.** Hertfordshire Sarcopenia Study (HSS) and extension (HSSe).

**Objective:**	To explore whether small size at birth is associated with cellular changes in human skeletal muscle that persist into adult life and have adverse consequences for muscle ageing in terms of muscle mass, lower strength and impaired metabolism.
**Methodology:**	Home visit followed by detailed clinical assessments during a day visit at the Wellcome Trust Clinical Research Facility, Southampton General Hospital ^21^.
**Key data availability:**	
• Cigarette smoking • Alcohol consumption • Short food frequency questionnaire (HSSe only) • Falls • Strawbridge frailty score • Fried frailty components • Townsend disability scale • Physical activity questionnaire • SF-36 health related quality of life • Co-morbidity • Current medications • Cognitive function (AH4 intelligence quotient, Mill Hill vocabulary test) • Day and night salivary stress hormones • Venous blood samples after overnight fast: glucose insulin HbA1c hormonal, inflammatory and DNA analyses • Biopsy of the *vastus lateralis* muscle using a Weil Blakesley conchotome • Immunohistochemical analysis of muscle: myofibre type, number, density and area • Muscle tissue stored at -80C for gene expression studies • DXA scan to quantify regional and total lean mass, fat mass and bone mineral content (Hologic Discovery) • Sub-maximal cycle ergometry exercise test (heart rate, energy expenditure and oxygen consumption) • Height; weight; waist, hip, mid upper arm, and thigh circumferences • Triceps, biceps, subscapular and suprailiac skinfold thicknesses (Crymych callipers) • Blood pressure (DASH 3000 device) • ECG • Grip strength (Jamar hand-grip dynamometer) • Timed 6m up-and-go test and 3m walk • Chair rises • Timed one-legged stand • 7-day wrist-worn Geneactiv physical activity monitoring	

**Table 8.** Hertfordshire Cohort Study component of the European Study of Osteoarthritis (EPOSA).

**Objective:**	To identify risk factors for, and consequences of, osteoarthritis (OA) for individuals and society and to explore cross-country differences in these patterns.
**Methodology:**	Collaborative study with five other centres in Germany, Holland, Sweden, Spain and Italy ^30^. HCS fieldwork conducted by a nurse home visit followed by x-rays at Hertford County Hospital. HCS EPOSA participants were recruited from men and women who had previously participated in the musculoskeletal follow-up study.
**Key data availability:**	
• Osteoarthritis questionnaire (affected sites, functional limitation, clinical procedures, joint replacement surgery) • WOMAC hip and knee pain, stiffness and function scores • Oxford hip and knee pain and limitation scores • Auscan pain, stiffness and function scores • EuroQoL EQ-5D quality of life (mobility, self care, activities, pain, anxiety, health) • Use of assistive devices • Falls • Fractures • Strawbridge frailty score • Fried frailty components • Townsend disability scale • Current medications • Cigarette smoking • Alcohol consumption • Household composition • Housing tenure • Short food frequency questionnaire • Nutrient intake from dietary supplements • Longitudinal Ageing Study Amsterdam physical activity questionnaire (LAPAQ) • Health service use (hospitalisations, operations, GP and health professional consultations) • Formal and informal home care • Mobility problems • Co-morbidity • Mini mental state examination (MMSE) • Height; weight; waist, hip and calf circumferences • Clinical hand examination for pain, • swelling and tenderness • Clinical hip and knee examination for flexion, rotation, pain, swelling and tenderness • Timed balance tests • Timed 6m up-and-go test and 8 foot walk • Chair rises • Grip strength (Jamar hand-grip dynamometer) • Lubben social network score • Pearlin mastery scale score • Maastricht Social Participation Profile (MSPP) scores • Pittsburgh Sleep Quality questionnaire (sleep duration, difficulties, disturbances) • Anxiety and depression (HAD) scores • 14-day pain calendar • X-ray of hip; antero-posterior and lateral x-rays of both knees (Kellgren/Lawrence grading scores, osteophytes, joint space and sclerosis)	

**Table 9.** Hertfordshire Cohort Study bone studies: 2012 and 2017.

**Objective:**	DXA scanning is a well-established technique for assessment of bone mass and areal bone density. However, newer technologies enable a more comprehensive understanding of bone health. Peripheral quantitative computed tomography (pQCT) provides a non-invasive assessment of bone strength and a volumetric assessment of bone density. In addition, a ‘virtual bone biopsy’ may be conducted by high resolution pQCT (HR-pQCT); this enables examination of the microstructure of separate compartments of bone (trabecular and cortical), bone geometry, and provides higher resolution imaging than standard pQCT. The objectives of the 2012 and 2017 HCS bone studies were: to use pQCT and HR-pQCT scanning to comprehensively, and longitudinally, characterise bone health; to explore the early life and environmental determinants of bone health in later life; and to investigate the interplay between muscle mass, strength and function and bone health.
**Methodology:**	Face to face questionnaires and scans at the MRC Elsie Widdowson Laboratory (Cambridge) among men and women who previously participated in the musculoskeletal follow-up study.
**Key data availability:**	
*2012* • Height and weight • DXA scan (whole body, proximal femur and lumbar spine) (Lunar Prodigy scanner) ** • Radial and tibial pQCT scans (Stratec XCT 2000XL instrument) • Distal radius and tibia HR-pQCT scans (Xtreme HR-pQCT scanner) ** • Jumping mechanography (single two-leg jump on Leonardo Ground Reaction Force Plate; muscle force, power, velocity and jumping height) *2017* • Height and weight • iDXA scan (whole body, proximal femur and lumbar spine) (GE-Lunar scanner) ** • Radial and tibial pQCT scans (Stratec XCT 2000XL instrument) • Timed balance tests • Timed 6m up-and-go test and 8 foot walk • Chair rises • Grip strength (Jamar hand-grip dynamometer) • Medical history and current medications • Falls and fractures • Townsend disability scale • Cigarette smoking and alcohol consumption • Marital status and household composition • Short food frequency questionnaire • EuroQoL EQ-5D quality of life (mobility, self care, activities, pain, anxiety, health) • Longitudinal Ageing Study Amsterdam physical activity questionnaire (LAPAQ) • Utilisation of health and social care services in the past month • Hospitalisation, medical specialist consultations, day care use, and receipt of formal care at home, in past year	

**Table 10.** Hertfordshire Cohort Study Vertical Impact on Bone in the Elderly (VIBE) study.

**Objective:**	To characterise levels and patterns of physical activity in older people and to investigate how physical activity affects bone health, muscle strength, physical capability, and osteoarthritis.
**Methodology:**	Participants completed a postal questionnaire. Wearable physical activity accelerometers were delivered and returned by post. This study was conducted in collaboration with the University of Bristol.
**Key data availability:**	
• Marital status • Highest educational qualification • Self-rated general health • Co-morbidity • Current medications • Smoking status • Alcohol consumption • Dietary dairy intake • Fried frailty self-reported components • Warwick-Edinburgh Mental Well-being Scale • Physical activity in the past 7 days • Physical activity previously throughout life • Fracture history since 45 years of age • Falls history in the past year • Falls Efficacy Scale fear of falling • Use of a walking aid • History of joint replacement • Reproductive history (women only) • Household composition • Housing tenure • Internet access at home • Sources of income (own and spouse/partner’s) • How well managing financially • Receipt of care (from household members or external sources) • SF-36 physical functioning scale • Townsend disability scale • Knee pain questionnaire • Life-Space Assessment (University of Alabama at Birmingham scale) • Short food frequency questionnaire • 7-day hip-worn triaxial accelerometer assessment of physical activity	

**Table 11.** Hertfordshire Cohort Study diet and physical activity focus groups.

**Objective:**	To better understand relationships between physical activity and diet in later life, and the factors that lead to deterioration in diet quality or change in habitual physical activity levels in later life.
**Methodology:**	In 2011, 443 HCS participants completed a food-frequency questionnaire during the EPOSA follow-up; this enabled comparison of their diet in 2011 with that reported by food-frequency questionnaire at HCS baseline approximately 10 years previously. We identified participants whose diets had remained stable or declined in quality; from these groups, we purposively sampled men and women to participate in to a series of focus groups designed to study influences on food choice and physical activity in later life. Eleven focus groups were held in Hertfordshire; the semi-structured moderator- led discussions were audio-recorded, transcribed verbatim and transcripts analysed thematically.
**Key data availability:**	
• Anonymised verbatim transcriptions of all discussions • Thematic analysis of emergent themes identified by inductive coding • Thematic map	

**Table 12.** Routinely collected data for Hertfordshire Cohort Study.

**a) Mortality**	
**Objective:**	To investigate the impact on cause-specific mortality of 1) early growth and 2) sociodemographic, lifestyle, medical and biological characteristics in later life. To monitor attrition among HCS members.
**Methodology:**	37,000 individuals born between 1911 and 1939 were identified from the HV records and flagged on the NHS Central Register for continuous notification of death (Objective 1). Sociodemographic, lifestyle, medical and biological data were collected from subsets of these individuals in later life, including the 3000 participants in HCS (Objective 2).
**Source:**	Office for National Statistics
**Key data availability:**	
• Date of death • Underlying cause of death (ICD-10) • Multiple causes contributing to death (ICD-10)	
**b) Hospital admissions**	
**Objective:**	To investigate risk factors for hospital admission among community dwelling older people.
**Methodology:**	An extract of Hospital Episode Statistics in-patient data covering the years 1998-2010 was obtained. 8741 admissions experienced by HCS members between the date of their home interview and 31/03/2010 were linked to the study database.
**Source:**	NHS Digital
**Key data availability:**	
• Postcode • Date of admission • Method of admission • Source of admission • Date of discharge • Destination on discharge • Main specialty • Primary diagnosis (ICD-10) • Other diagnoses (ICD-10) • Primary procedure (OPCS-4) • Other procedures (OPCS-4)	

***Predictors of osteoporosis.*** The Health Visitors’ records that are uniquely available in HCS have enabled investigation of the early-life origins of osteoporosis. Studies using DXA showed an increase in adult BMC with rising weight at birth, and more strongly, with weight at one year. Models that also included weight in adulthood showed impacts accruing from each time-point, with greater contributions of early growth to BMC than to BMD ^12^. In addition, growth during the first year was shown to alter the geometry of the adult hip ^13^. Studies using pQCT showed that bone strength was similarly related to early weights ^14^.

Risk of osteoporosis attributable to lifestyle has also been investigated in this cohort. While no social patterning has been observed ^15^, nor any relationship found between cigarette smoking, alcohol use, or physical activity and either BMC or BMD ^16^, alcohol has been associated with less radial cortical and trabecular bone in men and less tibial trabecular bone in women ^17^. Clustering of risk factors (physical activity, diet, alcohol consumption, smoking behaviour, grip strength, personal and family interactions, comorbidities) has been found to increase the risk of low BMD in women ^18^. Also in women, pQCT scans have shown that having a ‘healthier’ diet (characterised by greater consumption of fruit, vegetables and wholegrain cereals) is associated with increased bone area on pQCT scanning ^19^. Some lifestyle risk factors appear to interact with early life factors to influence osteoporosis risk, for example, in men, having a low birth weight and being a current smoker increased the risk of low femoral BMD ^16^.

***Osteoporosis and comorbidity.*** HCS has provided valuable insight into the interplay between osteoporosis and other chronic conditions. For example, in the case of cardiometabolic disease, analyses have shown that: BMD is associated with serum triglycerides and HDL ^22^; higher BMI is associated with lower 25(OH) vitamin D ^23^; arterial calcification affects the structure of cortical and trabecular bone in women, but not in men ^24^; hyperinsulinemia influences BMD through BMI ^25^; and the associations between lean and fat mass and bone microarchitecture differ ^26^. In the case of chronic inflammation in later life (inflammaging), HCS has shown that an elevated inflammatory state is associated with reduced BMD at baseline and with an accelerated rate of decline over time ^27^. Relationships between muscle and bone health are discussed in the section on sarcopenia that follows.

***Outcomes of osteoporosis.*** The primary outcome of osteoporosis is fracture, which carries high personal and societal costs. Risk of fracture has been elucidated in HCS, showing, for example, that history of falls adds to clinical risk factors and low BMD as a risk factor for fracture ^28^. Research using pQCT scans has shown that in women, cortical radial thickness and area, and tibial cortical area and density, are associated with adult fracture risk, and in men tibial SSI is associated with adult fracture risk ^29^. Finally, cluster analysis of bone microarchitecture from HR pQCT has demonstrated two separate phenotypes associated with high fracture risk ^31^.

### Osteoarthritis (OA)

OA is the most common joint disease affecting older people ^32^. It is an important element of the research programme of the LEU and its incorporated Arthritis Research UK MRC Centre for Musculoskeletal Health and Work. In HCS, radiographic, clinical and self-reported markers of OA have been collected at multiple time points ( Table 2– Table 12).

***Descriptive epidemiology.*** HCS data have been used to show that the method of ascertainment of OA status affects both its calculated prevalence ^33^ and the demographic characteristics with which it is associated ^34^. For example, prevalence of knee OA ranged from 18% for clinically diagnosed disease through 21% for self-reported disease to 42% when the diagnostic criteria were radiographic; with higher specificity and lower sensitivity for clinical and self-reported OA than for radiographically determined disease ^33^. Further work utilising data for multiple methods of ascertainment showed that in the presence of radiographically determined disease in the knee, signs and symptoms differed dependent on the bones involved, tibiofemoral OA being associated with both signs and symptoms, and patellofemoral OA only with clinical signs ^35^.

***Predictors of OA.*** Results from this study have demonstrated that growth before birth and in the first year both affect the likelihood of developing OA in later life ^36^. Specifically, an increase in number of osteophytes on hip radiographs has been associated with low birthweight and the number on knee radiographs with low weight at 1 year. An increased prevalence of clinical OA at the hand was also seen in those whose weight at 1 was low. Conversely, rheumatoid arthritis was not associated with early weight ^37^. The role of biochemical markers of bone in the aetiology of OA has also been investigated, finding that CTX-II and glucosyl-galactosyl-pyridinoline were associated with both osteophyte score and joint-space narrowing, whist CTX1 and osteocalcin were not ^38^.

***Outcomes of OA.*** The consequences of OA include an increase in the impact of objectively measured neighbourhood problems on participants’ physical activity ^39^ and quality of life ^40^. Pain is an important symptom of OA which, in HCS and other EPOSA cohorts, has been found to fully explain the association of OA with objectively measured (but not with self-reported) physical function ^41^. This suggests that individuals consider factors other than pain (e.g. comorbidity) in making an assessment of their physical function.

### Sarcopenia

Table 2– Table 12 show that HCS has collected a wealth of markers of the mass, strength, function and morphology of muscle. Together, these have enabled a rich body of research on the predictors and consequences of sarcopenia.

***Descriptive epidemiology.*** Sarcopenia may be defined by one or more of the markers collected in HCS, inter-relationships between which have been explored in detail ^42, 43^. The prevalence of sarcopenia across HCS, estimated according to European Working Group for Sarcopenia in Older People (EWGSOP) guidelines, but with lean mass estimated by skinfold measurements, was 4.6% in men and 7.9% in women ^44^. For subgroups of HCS in which imaging and anthropometric data coexist, estimates of prevalence have been calculated according to more than one operational definition of sarcopenia (EWGSOP; International Working Group on Sarcopenia; Foundation for the National Institutes of Health sarcopenia project; dysmobility syndrome), enabling the methods to be compared directly ^44, 45^. Late-life declines in muscle mass as well as strength have been observed using data from two of the time points shown on Figure 2 (MSFU and EPOSA); these data suggest that muscle mass declines less rapidly than strength or function ^46^.

***Predictors of sarcopenia.*** The early-life origins of sarcopenia have been investigated using data from the Health Visitors’ records. Weights at birth and one year of age have been related to adult anthropometry, showing that whilst BMI and lean mass rise with birthweight and with weight at one, fat mass has associations only with the latter ^47^. This suggests that whilst the prenatal environment affects lean mass, the postnatal environment is more influential in the development of adult adiposity. Direct measures of muscle size from pQCT scanning have confirmed that birthweight is associated with muscle area in the calf and forearm ^48^, whilst biopsies showed that smaller babies have lower total myofibre scores (kg/mm ^2^) than those who were bigger at birth ^49^. Grip strength was more strongly associated with birth weight than with growth during the first year of life in HCS participants ^50^. This again suggests that sarcopenia has primarily pre- rather than post-natal origins, though there is some evidence to link breast feeding during infancy with increased grip strength ^51^. A systematic review and meta-analysis of work in HCS and other cohorts showed that the positive relationship between birth weight and muscle strength exists across the lifecourse ^52^.

The effects of adult lifestyle on risk of sarcopenia have also been investigated. First, to examine the role of diet, associations of grip strength with specific nutrients, individual foods and dietary patterns were studied. Healthier diets were associated with higher grip strength, although the strongest association was with more frequent consumption of oily fish ^53^. Physical activity in old age, objectively measured by accelerometry, was found to be associated with decreased risk of EWGSOP sarcopenia and improved physical function, though not with grip strength ^54^. Grip strength was not associated with lifetime occupational exposure to physically demanding activities ^55^, a somewhat surprising finding in the light of evidence of social patterning in sarcopenia ^56^. The role of adult heath in causal paths to sarcopenia has also been investigated, identifying multimorbidity ^57^, inflammaging ^58^, and some ^59^, but not all ^60^, cardiovascular drugs as important influences.

***Outcomes of sarcopenia.*** Sarcopenia has profound consequences for health and wellbeing in later life and is associated in HCS with increased risk of hospital admission ^61^. Changes in ageing muscle have been shown to contribute to the metabolic syndrome and all its components ^62^; to self-rated health and health-related quality of life ^63^; and to bone mineral content ^64^. Together with strong independent relationships between muscle and bone size/strength ^65^, this latter finding supports the existence of a muscle/bone unit and provides some insight into the mechanisms that underlie it.

### Diet

***Determinants of diet quality.*** The HCS has described marked differences in dietary choices in later life. For example, healthier diets have been found to be associated with being female, reporting a non-manual social class, and with being a non-smoker ^66^. Psychological and social factors have important influences ^67^, greater involvement in social activities being associated with slower declines in diet quality ^68^. In addition, a relationship has been described between infant feeding and health behaviours in later life, such that people who were breastfed as infants were more likely to have healthier diets ^69^.

***Outcomes of diet quality.*** Our studies have described links between differences in nutrient intake and physical functioning in older women ^70^, and shown that there is a graded increase in the prevalence of poor physical functioning in older adults as the number of risk factors (from obesity, smoking and poor diet) increases ^71^. Less healthy diets also contribute to risk of hospital admission ^72^. Additionally, higher processed meat consumption and antioxidant intake have been related to poorer lung function in HCS ^73^, while low dietary antioxidants were also associated with poorer glucose tolerance ^74^.

***Methodology.*** Assessment of diet in older populations can be burdensome. In HCS, a short food frequency questionnaire was therefore developed and validated to assess compliance with a healthy dietary pattern in older community-dwelling adults ^75^.

## Routinely collected data

The Medical Research Council ^76^ and the Wellcome Trust ^77^ have advocated for the scientific and interdisciplinary potential of cohorts to be enhanced through linkage to routine health records and administrative datasets. Such linkage is particularly valuable in an ageing cohort like HCS, where attrition may lead to bias if data collection requires participant contact. Successful linkage has been achieved in HCS with mortality and Hospital Episode Statistics (HES) data ( Table 12), though difficulties persist over data access due to constantly changing regulations.

### Mortality

In total, the 1911 to 1939 Hertfordshire birth cohort comprises 37 000 men and women, 7916 of whom had died by the end of 1999. Risk of death from circulatory disease was lower among men and women who were heavier at birth. Women who were heavier at birth also had lower risk of death from pneumonia, injury, diabetes, and musculoskeletal disease ^78^.

### Hospital care

HCS was one of the first English cohorts to link with HES data and produced early evidence on the descriptive epidemiology of hospital admissions among older individuals ^10^. Such individual level data contrast with published statistics, which are at the population level. A novel methodology was developed to explore risk factors for admission in these multiple-failure survival data ^79^, demonstrating links between admission and grip strength ^61^, and showing that poor lifestyle risk factors have a cumulative effect on likelihood of admission ^72^.

## Genetics and epigenetics

Since its inception, HCS has studied the genetics of musculoskeletal ageing. The scientific approach has evolved across the years, alongside dramatic changes in the technologies available to characterise the genome. Early studies of genetic influences on adult disease in HCS considered the role of single nucleotide polymorphisms (SNPs) and their specific interaction with early life phenotypes as predictors of muscle and bone health in later life ^80, 81^. Haplotype studies followed ^81– 83^, and more recently HCS has been an important contributor to many genome wide association studies conducted by worldwide consortia ^84, 85^. Finally, in the Hertfordshire Sarcopenia Study (HSS), participation in the industry funded MEMOSA (Multi-Ethnic MOlecular determinants of human SArcopenia) collaboration has permitted cutting edge scientific techniques such as deep sequencing of RNA as well as high coverage methylation arrays to be completed in a subset of participants. Furthermore, muscle cells obtained from biopsies of older men and women have been cultured *in vitro* providing a biobank that together with RNA and methylation data, will permit the investigation of genetic and epigenetic pathways in musculoskeletal ageing. This is an exciting area for future research in HCS

## National and international collaborations

The HCS research group has a long and successful history of conducting collaborative research; principal local, national and international collaborators are detailed in Table 13. The nature of HCS collaborations varies widely, all are welcomed by the HCS research team. For example, bespoke results from statistical analyses and/or datasets have been provided to collaborators (ranging from PhD students to established research groups and international consortia) for inclusion in systematic reviews and meta-analyses. In other instances, students have visited the MRC LEU and worked alongside the HCS research team to conduct a specific sub-study or to run statistical analyses and draft a paper for publication. Another mode of collaboration is the provision of biological samples (e.g. plasma or DNA) to research groups and their laboratories; the information that they generate is returned to the HCS research team for inclusion in the master HCS databases. The resulting enhanced database is either analysed by HCS statisticians or released to the collaborators for them to analyse; either approach results in a jointly authored peer reviewed publication. Finally, the HCS research team may collaborate by submitting an ethics application jointly with a research group and conducting the study together; for example, the HPAT physical activity trial, the wellbeing follow-up study, and the VIBE study were conducted in collaboration with the MRC Epidemiology Unit (University of Cambridge), the MRC Unit for Lifelong Health and Ageing (at UCL) and the University of Bristol, respectively (see Figure 2 for timeline of HCS sub-studies).

**Table 13.** Location of principal Hertfordshire Cohort Study collaborators: local, national and international.

**Southampton**	
Arthritis Research UK/MRC Centre for Musculoskeletal Health and Work	University of Southampton
Centre for Research on Ageing	
Clinical and Experimental Sciences Academic Unit	
Human Genetics Research Division	
Institute of Human Nutrition	
NIHR Southampton Biomedical Research Centre	
University Geriatric Medicine	
Wellcome Trust Clinical Research Facility, Southampton General Hospital	
**UK**	
Epidemiology and Biostatistics	Imperial College London
National Heart and Lung Institute	
Respiratory Epidemiology and Public Health Group	
Department of Twin Research and Genetic Epidemiology	King's College London
GSK, Brentford	Middlesex
Musculoskeletal Research Group, Institute of Cellular Medicine	Newcastle University
Health Services for Older People	Nottingham City Hospital
MRC Unit for Lifelong Health and Ageing	University College London
MRC Centre of Epidemiology for Child Health	
Institute of Child Health	
Institute of Education	
Centre for Paediatric Epidemiology and Biostatistics	
Department of Epidemiology and Public Health	
Cohort and Longitudinal Studies Enhancement Resources (CLOSER)	
Arthritis Research UK/MRC Centre for Musculoskeletal Ageing Research Institute of Inflammation and Ageing	University of Birmingham
Musculoskeletal Research Unit	University of Bristol
School of Social and Community Medicine	
MRC Epidemiology Unit	University of Cambridge
MRC Elsie Widdowson Laboratory	
Early Growth Genetics (EGG) Consortium	
Section of Ageing and Health	University of Dundee
NIHR Oxford Biomedical Research Centre	University of Oxford
The Nuffield Department of Orthopaedics, Rheumatology and Musculoskeletal Sciences (NDORMS)	
Institute of Musculoskeletal Sciences, The Botnar Research Centre	
Oxford Centre for Diabetes, Endocrinology and Metabolism (Early Growth Genetics Consortium, EGG)	
Wellcome Trust Centre for Human Genetics (GIANT Consortium)	
MRC Social & Public Health Sciences Unit	University of Glasgow
SpiroMeta consortium, Departments of Health Sciences and Genetics	University of Leicester
Arthritis Research UK Centre for Epidemiology	University of Manchester
Academic Rheumatology	University of Nottingham
Arthritis Research UK Pain Centre	
Metabolic Disease Group	Wellcome Trust Sanger Institute
**International**	
University of Queensland	Australia
Flinders University	
School of Human Sciences, Faculty of Science, University of Western Australia	
Department of Public Health, Epidemiology and Health Economics, University of Liege	Belgium
Division of Geriatric Medicine, Jewish General Hospital/McGill University	Canada
Zhejiang University	China
INSERM Research Unit	France
Department of Rheumatology, Lille University Hospital	
Department of Medicine and Therapeutics, Chinese University of Hong Kong	Hong Kong
Department of Medicine, University of Padova (European Project on OSteoArthritis, EPOSA)	Italy
National Research Council, Neuroscience Institute - Aging Branch, Padova	
University of Tokyo	Japan
University of Auckland	New Zealand
Department of Public Health, Norwegian University of Science and Technology	Norway
Department of Medical Epidemiology and Biostatistics, Karolinska Institutet (Meta-Analyses of Glucose and Insulin-related traits Consortium, MAGIC)	Sweden
Atherosclerosis Research Unit, Department of Medicine Solna, Karolinska Institutet	
Department of Ageing and Life Course, World Health Organization, Geneva	Switzerland
Nestlé Institute of Health Sciences, Swiss Federal Institute of Technology, Lausanne (MEMOSA and EPIGEN collaborations)	
Erasmus Medical Center, Rotterdam (TREAT-OA consortium)	The Netherlands
Genetic Laboratory Department of Internal Medicine, Erasmus Medical Center, Rotterdam	
Department of Epidemiology and Biostatistics, University Medical Center, Amsterdam (European Project on OSteoArthritis, EPOSA)	
Department of Neurology, Academic Medical Centre (ICARA project), University of Amsterdam	
Department of Biostatistics, Boston University	USA
Harvard University (BIG collaboration on cardiometabolic disease)	
John Hopkins University	
Broad Institute of Harvard and Massachusetts Institute of Technology, Meta-Analyses of Glucose and Insulin-related traits Consortium (MAGIC)	
Division of Epidemiology, Human Genetics, and Environmental Sciences, University of Texas (the CHARGE whole grain foods study group)	
Division of Cardiology, Massachusetts General Hospital, Boston	
Framingham Heart Study, National Heart, Lung, and Blood Institute (NHLBI)	
Department of Biostatistics, University of Michigan (Genetic Investigation of ANthropometric Traits Consortium, GIANT)	

## The future

HCS is a flagship cohort that has demonstrated lifecourse influences on musculoskeletal health in later life. All of the information collected to date is extensively documented and carefully curated and, as evidenced by a broad body of collaborators and over 250 peer-reviewed publications to date, the cohort will continue to be an invaluable resource for national and international research on the lifecourse determinants of musculoskeletal health in later life. Cohort members remain flagged with NHS Digital for ongoing notification of death, and further extracts of HES data would enable long-term follow-up of morbidity for the whole cohort (subject to funding and permission to access data). 

Two examples of future research areas in HCS are: first, development of lifecourse interventions to promote maintenance of health, and slow loss of function, of the musculoskeletal system in later life; and second, investigation of the mechanisms that explain how early life environment influences health in later life.

The ambition to develop lifecourse interventions to maintain the health of the musculoskeletal system in later life is a natural progression from the epidemiological studies that have been conducted in HCS and other cohorts to date. Diet and physical activity are now well established as key influences on musculoskeletal health in later life, but research in HCS and elsewhere has shown that lifestyle risk factors cluster together to impact on physical function in later life ^71^. Therefore, any lifestyle based intervention strategy would be well advised to adopt a holistic whole-person approach. Accordingly, the HCS research team’s objective is to develop and test the feasibility of a combined healthy conversation and physical activity intervention to promote muscle mass, strength and function in later life with the ultimate aim of preventing falls and fractures. If this is successful, the intervention will be implemented more widely and its efficacy evaluated.

Epigenetics is an important area for research that seeks to move from epidemiological associations to identification of the mechanisms that underpin early life influences on health in later life. Epigenetics is the study of changes in organisms caused by modification of gene expression rather than alteration of the genetic code itself. For example, in HSS and HSSe participants, we are in the early phase of investigating whether the expression of genes associated with cellular pathways is altered during muscle ageing and how this contributes to sarcopenia. We are also studying genes associated with inflammation, mitochondrial function and the regulation of muscle growth, and taking the novel approach of investigating epigenetic pathways by studying DNA methylation markers in muscle tissue and also in cultured primary skeletal muscle cells. The results from this work will give us clues about how sarcopenia begins, progresses, and what can be done to prevent or treat it.

Epigenetics underpins the ambition to expand HCS to an intergenerational study. The theory of transgenerational epigenetics implies that maternal environment during pregnancy transmits to offspring and affects their subsequent gene expression ^86^. An intergenerational study in HCS is a very special opportunity to investigate how epigenetic expression transmits across generations, and indeed to explore commonalities and differences in musculoskeletal health across generations. To date, more than 1,000 members of the HCS cohort (the ‘F0 generation’) have provided us with the names and contact details of their children (the ‘F1 generation’) and grandchildren (the ‘F2 generation’). We are now in the process of recruiting these children and grandchildren to an HCS three generation cohort (HCS 3G); in the first instance we are seeking their consent to contact them in the future with invitations to participate in specific studies as they are planned. To date, more than 700 children and grandchildren have agreed to be members of the HCS 3G study. Following on in the tradition of their parents or grandparents who have participated so generously in HCS, these people will make an important contribution to our understanding of lifecourse and intergenerational influences on musculoskeletal health. 

## Data availability

Hospital Episode Statistics and mortality data were obtained from NHS Digital under Data Sharing Agreements numbered 148284 and 343023; but cannot be made openly available for ethical reasons. HCS data collected directly by the MRC LEU are accessible via collaboration with the HCS research group as described above. Initial enquires should be made to Professor Cyrus Cooper (Principal Investigator, e-mail: **cc@mrc.soton.ac.uk**). Potential collaborators will be sent a collaborators’ pack and asked to submit a detailed study proposal to the HCS Steering Group; applications will be reviewed at the first available steering group meeting (held three times annually).
