# Supplementary material for: The Hertfordshire Cohort Study: an overview
Source: F1000Res. 2019 Jan 21;8:82. [Version 1] doi: 10.12688/f1000research.17457.1 (PMC6381804; doi:10.12688/f1000research.17457.1)
Supplement: Peer review history

# Peer review: Reviewer response for version 1

**DOI:** 10.5256/f1000research.19091.r43326

**Published:** 2019-02-22

**Authors:** Cecily Kelleher

**Affiliations:** 1 College Of Health And Agricultural Sciences, University College Dublin, Dublin, Ireland

This report provides an overview of the Hertfordshire Cohort Study, one of the most influential of epidemiological studies which provided ground-breaking evidence of early life influences on the development of adult chronic disease and laid the foundation for a huge corpus of scientific work on the developmental origins of health and disease hypothesis. The original retrospective cohort study was established by linking contemporary health outcome data to health visitor records collected between 1911-1930 in the English county of Hertfordshire.

 The paper concentrates on the highly innovative strategy of further follow-up examinations and data collection in the younger cohort of 3000 men and women who were born between 1931 and 1939. The objective was to evaluate interactions between intra-uterine and early postnatal environment, adult diet and lifestyle, genetics in the aetiology of chronic disorders of ageing including osteoporosis. A comprehensive battery of social, biological and health status variables was collected and the summary of studies carried out between 1998-2018 is summarised in Figure 2. A further highly original development is the inter-generational follow-up now planned and in train. To date more than 1000 members have provided contact details for their children and grandchildren, which will make this one of very few prospective studies with three generation follow-up. There is also detailed linkage to routine health records and administrative datasets, mortality and hospital episodes statistics.

 The review provides a comprehensive summary of key publications in the areas of osteoporosis, osteoarthritis and diet and this study has greatly added to our understanding of the aetiology and natural history of these outcomes. There are also a number of detailed tables which summarise the wealth of data collected at different stages of the study.

 My comments are all minor and primarily to do with presentation of the data in the report. Firstly, as a suggestion, I think the report would flow better if the sections on diet, routinely collected data, genetics and epigenetics and possibly National and International collaborations (currently pages 15-16) were moved back earlier to precede the outcome sections, focusing particularly on Osteoporosis, Osteoarthritis, Sarcopenia and Diet (currently pages 4-15). It might make the scope of the study clearer to the general reader. Also, the tables provide considerable detail and if the variables were sequenced in the same order and as appropriate sub-categorised, it would be easier to cross-reference what was collected and when.

I have read this submission. I believe that I have an appropriate level of expertise to confirm that it is of an acceptable scientific standard.

# Peer review: Reviewer response for version 1

**DOI:** 10.5256/f1000research.19091.r43324

**Published:** 2019-02-18

**Authors:** Aravinda Guntupalli

**Affiliations:** 1 Public Health, Open University, Milton Keynes, UK

The paper provides an excellent overview. See below a few comments that could further improve the overview. 

**1. Motivation and contribution of the paper**

 The Hertfordshire cohort study has resulted in several important discoveries related to the relationship between conditions in infancy and later life and this paper summarises the outcomes very well.

 However, I believe that it would be important to further highlight the motivation of the paper in the introduction. Most importantly, the following questions should be addressed:

1. What can we learn from the Hertfordshire cohort study (three most prominent findings)? 
2. Can the experience in setting up and conducting the Hertfordshire cohort study be transferred to other settings (put differently are there country and context specific circumstances that need to be taken into consideration)?
3. What are the main challenges (perhaps costs) that need to be considered?

Hence, it would be important to provide a clear motivation for this paper before discussing the historical background. Most importantly, the authors need to convince potential readers, who maybe unfamiliar with the UK, why this paper and the experience gained in conducting the Hertfordshire cohort study matters for their research agenda or potential cohort studies. Put differently, can the success of the Hertfordshire cohort study be replicated in other countries?

**2. Age, period and cohort effect**

 The early study focused on men and women born between 1911-30 with a special focus on 1920 and 1930 cohort. The 1920-30 cohort were are born at the tail end of the 'Spanish flu' and the start of the great depression. The younger cohort (the cohort of this study) comprises of 3000 men and women born between 1931 and 1939. During this period the great depression impact continued and the World War 2 started. It would be good to see the differences in findings between these two older and newer cohort studies from this context, if it is possible. Also, it would be good to say something on the socio-economic situation of the 1931-39 cohort. Would any of the historical events impact the results perhaps through survival bias? How was the infant mortality in this period? Can you describe the socio-economic status of the cohort and the differences in the ageing experiences by SES, if such information is available? 

**3. Methods of data collection over time**

 The paper discusses various methods of data collection summarised in Table 2. It would be interesting to know whether these methods (e.g. home interviews, clinic visits) have changed over time due to improved technology (e.g. home testing kits, questionnaires via mobile phones or email links etc.). Finally, could the authors provide cost estimates per participant? This would be valuable information for other research projects that consider setting up a cohort study in other countries and settings.

**4. The ‘early studies’ versus the Hertfordshire cohort study**

 The authors highlight that the ‘early studies’ had limitations, which were subsequently addressed. It might be useful to explain in detail what the limitations were. What can readers learn from this experience?

I have read this submission. I believe that I have an appropriate level of expertise to confirm that it is of an acceptable scientific standard.

# Response

**Published:** 2019-02-20

**Authors:** Cyrus Cooper

**Affiliations:** MRC Environmental Epidemiology Unit, University of Southampton, UK

We thank Dr Guntupalli for her positive assessment of our manuscript. Her comments are interesting, particularly her suggestion about placing the cohort in its broader historical and socioeconomic context. Information has previously been published (in papers and books) which does serve this purpose but it would be useful to incorporate some detail with this particular manuscript (either by making minor changes to the text of the manuscript, or by posting specific responses to her comments via this online system). We will consider how best to provide further details.

# Peer review: Reviewer response for version 1

**DOI:** 10.5256/f1000research.19091.r43323

**Published:** 2019-02-12

**Authors:** Rebecca Reynolds

**Affiliations:** 1 Centre for Cardiovascular Science, Edinburgh Bioquarter, University of Edinburgh, Edinburgh, UK

This is a clearly written overview of the Hertfordshire Cohort Study. The manuscript presents the background leading to the start of the Cohort and then logically describes the study phases and methods with clear summary tables of the variables collected. The manuscript reports key outcomes in some of the major areas of research that have been conducted using the Cohort, particularly in relation to bone and musculoskeletal health and diet. Details of linkage to routinely collected data, contribution to genetic and epigenetic studies and key collaborators are listed.  Plans for the future directions of the cohort, including recruitment to an intergenerational study are described. Overall, the manuscript provides a very useful reference tool and resource for parties interested in collaborating with the Southampton team. My only suggestion for improvement of the manuscript would be to add a paragraph on the limitations of the Cohort e.g. whether there are variables that are not available or with missing data, and whether the lack of these data means that there are certain study questions that cannot be readily addressed using the Cohort. For example, it does not appear that pregnancy-related variables are available in the original cohort, but I presume some may be available in the new intergenerational cohort?

I have read this submission. I believe that I have an appropriate level of expertise to confirm that it is of an acceptable scientific standard.

# Response

**Published:** 2019-02-20

**Authors:** Cyrus Cooper

**Affiliations:** MRC Environmental Epidemiology Unit, University of Southampton, UK

We thank Prof Reynolds for her positive assessment of our manuscript. We have taken her useful comments and suggestions on board and will consider how best to respond to them (either by making minor changes to the text of the manuscript, or by posting specific responses to her comments via this online system).
